# Supplementary material for: Exploring the Perceptions of Women from Under-Resourced South African Communities about Participating in a Low-Carbohydrate High-Fat Nutrition and Health Education Program: A Qualitative Focus Group Study
Source: Nutrients. 2020 Mar 25;12(4):894. doi: 10.3390/nu12040894 (PMC7231178; doi:10.3390/nu12040894)
Supplement: Supplementary file 1 [file nutrients-12-00894-s001.pdf]

File S1

<u>Focus Group Discussion semi structured questions.</u>

1. Thinking back over the time, what was the major influence on your decision to participate in the EBSA program?
2. What did you think of the educational sessions during the program?
    a. How easy were these sessions to understand?
    b. What aspects did you like or were useful?
    c. Which aspects didn't you like or were not useful?
3. How did you feel about the number and frequency of the sessions?
4. What influenced your attendance to the sessions of the EBSA program?
    a. What made it difficult to attend all the sessions?
    b. What made it easy to attend all the sessions?
5. What are your thoughts on the diet that EBSA recommended during the program?
    a. What were some of the things that helped you follow the diet?
    b. What were some of the challenges you faced?
6. After the program, how did you feel about following the recommended diet?
    a. Can you think of the most difficult food to get hold of or eat from the recommended foods?
    b. Can you think of the easiest food to get hold of or eat from the recommended foods?
    c. What can the EBSA program do to better support the change of diet?
7. How did people around you support you while doing the EBSA program?
    a. How did your family/friends/colleagues support/not support you?
8. In your experience, how did the EBSA program affect your life?
    a. Were there any positive impacts?
    b. Were there any negative impacts?
9. What do you think could be improved from the EBSA sessions?
10. What do you think about doing another EBSA program in your area?
